# Oxygen Reduction Activity and Stability of Composite Pd_x_/Co-Nanofilms/C Electrocatalysts in Acid and Alkaline Media

**DOI:** 10.3389/fchem.2018.00596

**Published:** 2018-11-29

**Authors:** LuLu An, Yumei Chen, Jianchao Shi, Jianliang Cao, Baozhong Liu, Juan Yang

**Affiliations:** Department of Energy and Chemical Engineering, College of Chemistry and Chemical Engineering, Henan Polytechnic University, Jiaozuo, China

**Keywords:** composite electrocatalyts, palladium nanoparticles, co nanofilms, oxygen reduction reaction, alkaline solution, acid solution

## Abstract

The morphology tuning of Pd and Pd-M nanoparticles is one of the significant strategies to control the catalytic activity toward oxygen reduction reaction (ORR). In this study, composite Pdx/Co-nanofilms/C electrocatalysts of Pd nanoparticles implanted onto Co nanofilms were synthesized on an immiscible ionic liquid (IL)/water interface for ORR. The Pd nanoparticles implanted onto Co nanofilms show a marked distortion of crystal lattice and surface roughness. These Pdx/Co-nanofilms/C electrocatalysts exhibit enhanced activity for ORR compared with Pd/C and PdxCo/C catalysts in both acid and alkaline solutions, in which the Pd3/Co-nanofilms/C catalyst displays the highest ORR mass activity. The superior ORR mass activities of the fabricated Pdx/Co-nanofilms/C catalysts may be mainly attributed to their larger catalytic areas, which are conferred by the rough surface of Pd nanoparticles with a distorted crystal lattice, and the synergistic effect between the surface Pd atoms and the 2D Co nanofilm substrate. The relationship between ORR mass activity and Pd/Co atom ratio varies in different electrolytes. Furthermore, by using proper heat-treatment methods, the Pdx/Co-nanofilms/C catalysts exhibit improved cycling stability compared with pure Pd/C catalyst after extended potential cycling.

## Introduction

Development of low-cost, efficient and stable electrocatalysts for oxygen reduction reaction (ORR) is one of the key factors to promote the commercialization of low-temperature fuel cells (Shao et al., 2016; He et al., 2017; Sui et al., 2017; Wang et al., 2018). Pd-based electrocatalysts have attracted considerable attention in research for non-Pt catalysts for ORR, because Pd has a similar structural characteristic to Pt, is relatively abundant on earth and displays the four-electron reduction of oxygen (Zhen et al., 2014; Doan et al., 2016; Zhang et al., 2016). So far, the most popular strategies including alloying Pd with various transition metals [e.g., Co (Wei et al., 2011; Rahul et al., 2014; Yun et al., 2015), Fe (Holade et al., 2016; Bampos et al., 2017), Au (Kuttiyiel et al., 2014; Wang Y. et al., 2016)] and Cu (Zheng et al., 2015; Wang G.W. et al., 2016) and choosing optimized support materials or heterostructural nanocomposites, such as carbon materials [e.g., XC-72, Graphene (G) and Reduced graphene oxide (RGO)] (Liu et al., 2015b; Yun et al., 2015; Zheng et al., 2015; Song et al., 2016), metal oxides (e.g., W_18_O_49_ and TiO_2_) (Maheswari et al., 2013; Lu et al., 2014), and transition metal carbides (e.g., Co_6_Mo_6_C_2_, Co_3_W_3_C, and Fe_2_MoC) (Li et al., 2014; Yan et al., 2015), have been applied to develop more active and durable ORR electrocatalysts. The enhanced ORR activities of these Pd-based electrocatalysts are believed to be due to the electronic structure modification of Pd by the alloying elements through the so-called strain and ligand effects or the electron transfer between the support materials and Pd nanoparticles named synergistic effect. For instance, alloying Pd with a transition metal with a smaller atom distance (e.g., Co, Ni, and Fe), the Pd–Pd interatomic distance decreases compared with pure Pd in the optimal alloy ratios (Wei et al., 2011; Rahul et al., 2014; Zhen et al., 2014; Yun et al., 2015; Bampos et al., 2017). Such a compressive strain of Pd–Pd causes a downshift of the d-band center of surface Pd, which consequently enhances the ORR activity through weakened bonding of oxygenated species, such as OH_ads_. As well, the nanocomposites of Pd nanoparticles deposited on bimetallic carbide support materials (e.g., Co_6_Mo_6_C_2_, GC-Fe_2_MoC) show superior activity and stability for ORR in acid media. The excellent performance of Pd/bimetallic carbide catalysts may be attributed to the excellent electron-donating (synergistic effect) of bimetallic carbide to Pd, which not only facilitates the reduction of oxygen but also increases the linkage strength between Pd and bimetallic carbide (Li et al., 2014; Yan et al., 2015).

Recent studies have manipulated the morphologies and surface structure of Pd and Pd-M alloy nanoparticles to control the catalytic activity toward ORR (Xiao et al., 2009; Lu et al., 2014; Poon et al., 2014; Zhen et al., 2014; Srejic et al., 2015). Lu et al. (2014) reported that strongly coupled Pd nanotetrahedron/tungsten oxide nanosheet hybrids exhibited not only surprisingly high activity but also superior stability for ORR in alkaline solutions. Such enhanced electrocatalytic activity and durability are associated with the increased number and improved catalytic activity of active sites, which is induced by the strong interaction between the Pd tetrahedrons and W_18_O_49_ nanosheet supports. Poon et al. (2014) showed that amorphous Pd nanoparticles synthesized by stepwise electroless deposition displayed superior ORR activity and stability compared with crystalline Pd nanoparticles and commercial Pt/C and Pd/C in KOH solution. Liu et al. (2015a) presented five-fold twinned Pd_2_NiAg nanocrystals with a Ni-terminal surface, which demonstrated excellent electrocatalytic performance for ORR in alkaline media better than that of commercial Pt/C catalysts. Such, the morphology tuning of Pd and Pd-M nanoparticles are still one of significant strategies to control the catalytic activity toward ORR. It is worth noting that a considerable amount of studies has proven that the ORR activities of Pd and Pd-based nanomaterials are comparable to that of Pt/C in alkaline media (Poon et al., 2014; Rahul et al., 2014; Zhen et al., 2014; Yun et al., 2015; Doan et al., 2016; Zhang et al., 2016; Bampos et al., 2017). Furthermore, Pd or Pd-based electrocatalysts possess predominant stability in alkaline solution owing to a less corrosive environment for the catalysts and electrodes in alkaline fuel cells.

Transition metal Co or Co-decorated catalysts have recently attracted considerable attention because of their low cost and promising applications in oxygen electrode (Cheng et al., 2017a,b; Su et al., 2017; Gong et al., 2018; Li et al., 2018). The Pd-Co alloy catalysts, in which the addition of transition metal Co could facilitate the dissociation of the O-O bonds () and thereby the produced Co-O_ads_ species might transfer to the Pd site to promote the electrochemical reduction of oxygen, have been reported to enhance the activity for ORR through adjusting the amount of Co alloyed, the Pd-Co particle size and the morphology (Wei et al., 2011; Arroyo-Ramírez et al., 2013; Antolini, 2014; Rahul et al., 2014; Yun et al., 2015; Huang et al., 2017). In the present study, Pd nanoparticles are implanted onto Co nanofilms to form Pd_x_/Co-nanofilms/C composite electrocatalysts on an immiscible ionic liquid (IL)/water interface through a sequential reduction approach. The assembly of Pd atoms is affected by 2D Co nanofilm substrate, and the Pd nanoparticles exhibit a distorted crystalline structure and surface roughness. Thus, prepared Pd_x_/Co-nanofilms/C catalysts have enhanced ORR catalytic activity in both acid and alkaline media. The designed Pd_x_/Co-nanofilms/C catalysts exhibit superior stability compared to Pd/C catalyst in alkaline media after proper heat-treatment. And the structure–activity relationship between the ORR mass activity and the structure of Pd/Co-nanofilms was discussed.

## Experimental

### Materials preparation and physical characterization

Pd_x_/Co-nanofilms/C electrocatalysts were synthesized including the use of an immiscible imidazolium-based IL to achieve controllable metal nanostructured materials (Pd nanoparticles, Co nanofilms) (Chen et al., 2014) on the immiscible IL/water interface and a two-step reduction procedure (Chen et al., 2016). Firstly, a mixture of IL (6.00 g) and KBH_4_ aqueous solution (20 mL, 50 mM) was stirred uniformly by ultrasonic assisted to form a highly dispersed, superfine IL/water interface solution and heated to 60°C. Another mixture of Co(Ac)_2_ aqueous solution (20 mL, 5 mM) and certain amount of XC-72 (mass ratio of Co+Pd to XC-72: 1/4) pre-mixed ultrasonically for 30 min was then added into the solution drop by drop. The reacting solution was stirred continuously for another 40 min to prepare Co-nanofilms/C. Secondly, the deposition of Pd nanoparticles onto Co nanofilms was reduced under 40°C with KBH_4_ (molar ratio of KBH_4_/PdCl_2_ = 10/1) as a reducing agent after rinsing PdCl_2_ (atom ratio of Pd/Co: 3:1 or 2:1) for 30 min in Co-nanofilms/C colloid system. The reacting solution was then cooled down to room temperature after stirring continuously for 3 h. The whole process was carried out under a N_2_ atmosphere. Finally, the resulting black powders were isolated by centrifugation and washed 3–4 times with ethanol. Thus-prepared catalysts were then dried at 30°C in a vacuum oven and stored for use. For comparison, Pd/C and Pd_x_Co/C alloy catalysts were prepared in the same synthesis system. Differently, After the first step to form the IL/water interface solution, the mixture of precursor salt aqueous solution (PdCl_2_ or Co(Ac)_2_ and PdCl_2_) and certain amount of XC-72 pre-mixed ultrasonically for 30 min was added to the solution drop by drop and stirring continuously for 3 h under 60°C. Pd and Pd_x_Co nanoparticles are deposited on XC-72 directly reduced by KBH_4_. The molar ratio of KBH_4_/PdCl_2_ (or KBH_4_/PdCl_2_+Co(Ac)_2_) = 10/1. IL: 1-octyl-3-methylimidazolium hexafluorophosphate [C_8_ min]PF_6_.

The morphological features of the prepared samples was measured using a JEOL JEM-2100FEF transmission electron microscope (TEM) operating at a 200 kV accelerating voltage. Power samples for TEM were dispersed in ethanol by ultrasonic dispersion for 30 min and a drop of suspension was deposited on copper grids coated with thin carbon film. The data of X-ray photoelectron spectroscopy (XPS) was obtained on A Kratos Ltd. XSAM-800 spectrometer and fitted using the software XPSPEAK41 (Shirley function as baseline, Gauss–Lorentzian linearity fitting).

### Electrochemical measurements

Electrochemical measurements were performed on a CHI760A potentiostat with a three-electrode configuration. The as-prepared electrode, a Pt foil and a saturated calomel electrode (SCE) were applied as the working electrode, counter electrode and reference electrodes, respectively. The working electrode was prepared by coating the catalyst ink onto a glassy carbon rotating disk electrode (RDE, geometric area: 0.196 cm^2^), in which accurately 5 mg catalyst was introduced into 1 mL solution of Nafion in isopropyl alcohol (0.05 wt%) sonicated for 30 min to form uniform ink.

The cyclic voltammogram (CV) test was conducted in Ar-saturated 0.5 M H_2_SO_4_ or 0.1 M NaOH solution at a scan rate of 50 mV/s. The steady-state polarization curves for ORR were recorded in O_2_-saturated electrolyte at a scan rate of 5 mV/s and a rotation rate of 1,600 rpm. The working electrode was first cleaned by potential cycling between 0.042 and 1.342 V in 0.5 M H_2_SO_4_ solution or 0.0103–1.5103 V in 0.1 M NaOH at 500 mV/s and the reproducible CVs should be obtained before each electrochemical test. Usually, the cyclic voltammograms (CVs) in the first few cycles change significantly. Even, the electrochemical areas gradually increase along with the potential scans. After 10–20 cycles of 500 mV/s, the successive cycling would give relatively reproducible CVs. It should be pointed out that “reproducible” here does not mean “no change.” Such an electrochemical polish is necessary to get rid of the surface contaminations of catalyst particles and this fast electrochemical activation cannot alter the original structure of samples. The stability is evaluated by measuring the cumulative change loss of their electrochemical surface area after long-term continuous cycling under accelerated potential region in Ar-saturated electrolyte with a scanning rate of 50 mV/s. The measurements were carried out at room temperature (27 ± 1°C). All potentials are reported with respect to RHE and the current densities in the CVs are normalized by the Pd loading of the electrode.

## Results and discussion

### Physical characterization

**Figure 1:**
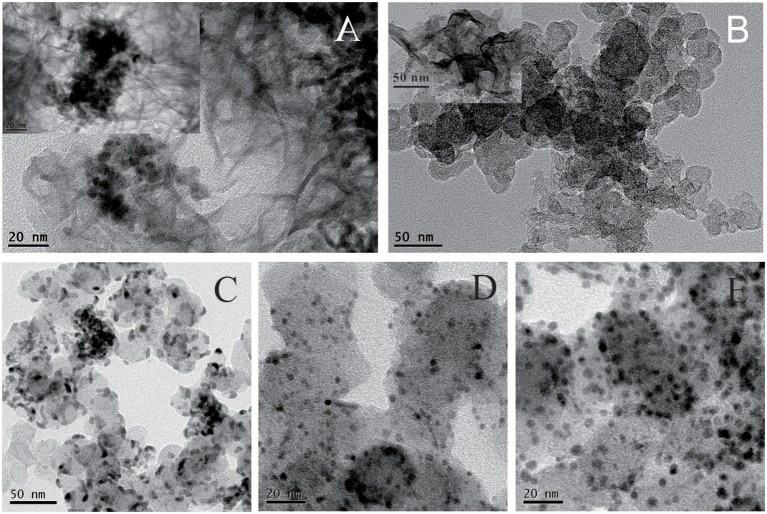
Representative TEM images of the prepared **(A)** Pd/Co-nanofilms materials, **(B)** the Co-nanofilms/C; TEM images of the prepared catalysts of **(C)** Pd/C, **(D)** Pd_2_Co/C, and **(E)** Pd_2_/Co-nanofilms/C.

**Figure 2:**
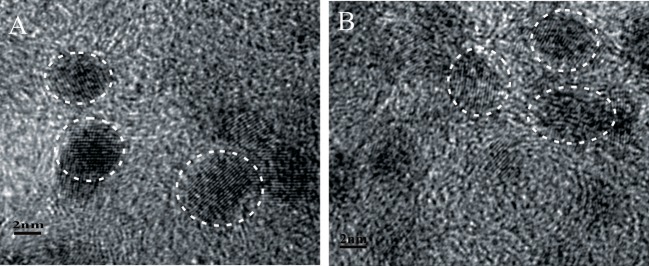
Representative HRTEM images of the catalyst particles of **(A)** Pd_2_Co/C and **(B)** Pd_2_/Co-nanofilms/C.

The fabrication of Pd_x_/Co-nanofilms/C catalysts in this study relies on an interfacial synthesis technique by the use of an immiscible IL/water interface to achieve controllable metal nanostructured materials (Pd nanoparticles, Co-nanofilms) (Chen et al., 2014) and a sequential reduction process to implant Pd nanoparticles onto Co nanofilms (Chen et al., 2016). The representative TEM images of the non-carbon supported Pd/Co-nanofilms show that Co atoms assemble to Co-nanofilms with edges curled into nanotubes, and almost all Pd nanoparticles, which are seriously aggregated, are preferentially impanted onto the Co-nanofilms (Figure 1A; also seen references Chen et al., 2014, 2016). To obtain highly dispersed Pd_x_/Co-nanofilms/C catalysts, the treatments including pre-mixing Co salt and XC-72 support uniformly by ultrasonic and rinsing Pd precursors for 30 min in the second reduction process, respectively, are applied to conquer aggregation through the onsite reduction to deposit Co nanofilms on the XC-72 support and heterogeneous nucleation to implant Pd nanoparticles preferentially onto the Co nanofilms evenly. The Figure 1B give the TEM image of the Co-nanofilm/C materials, the Co nanofilms are difficult to be identified in the most TEM images expect for very few edges. The TEM images in Figures 1C–E show that all of the prepared Pd, Pd_2_Co, and Pd_2_/Co-nanofilms particles have an average size of 5 nm and are relatively uniformly dispersed on carbon supports. High-resolution TEM (HRTEM) images of the selective catalyst particles of Pd_2_Co/C and Pd_2_/Co-nanofilms/C catalysts are shown in Figure 2. The composite materials of Pd_2_/Co-nanofilms exhibit a marked distortion of crystal lattice and a rough surface, which are different from the Pd_2_Co alloy particles mostly possessing a relatively fine crystalline structure with a smooth surface (observed in the particles signed by dashed circles). The Co nanofilms are also difficult to distinguish from the TEM and HRTEM images of Pd_2_/Co-nanofilms/C catalysts, possibly because that the existence of XC-72 supports destroy long-range IL/water interface, correspondingly that the Co atoms cannot form continuously complete nanofilms and the Co nanofilms may instead break into pieces (also seen reference Chen et al., 2016). The XRD responses of Pd/C, Pd_x_Co/C and Pd_x_/Co-nanofilms/C catalysts indicate that the diffraction peaks of Pd nanoparticles on Co nanofilms become broader compared with those of the Pd/C and Pd_2_Co/C catalysts and the diffraction peaks of separated Co nanofilms are not resolved on the XRD patterns of Pd_x_/Co-nanofilms/C catalysts (Chen et al., 2016). The XRD results confirmed that the pre-deposited 2D Co nanofilms affect the assembly of Pd atoms and Pd nanoparticles with more complex crystalline are formed onto Co-nanofilms/C, correspondingly to modulate the electronic properties or geometric structure of surface Pd atoms.

**Figure 3:**
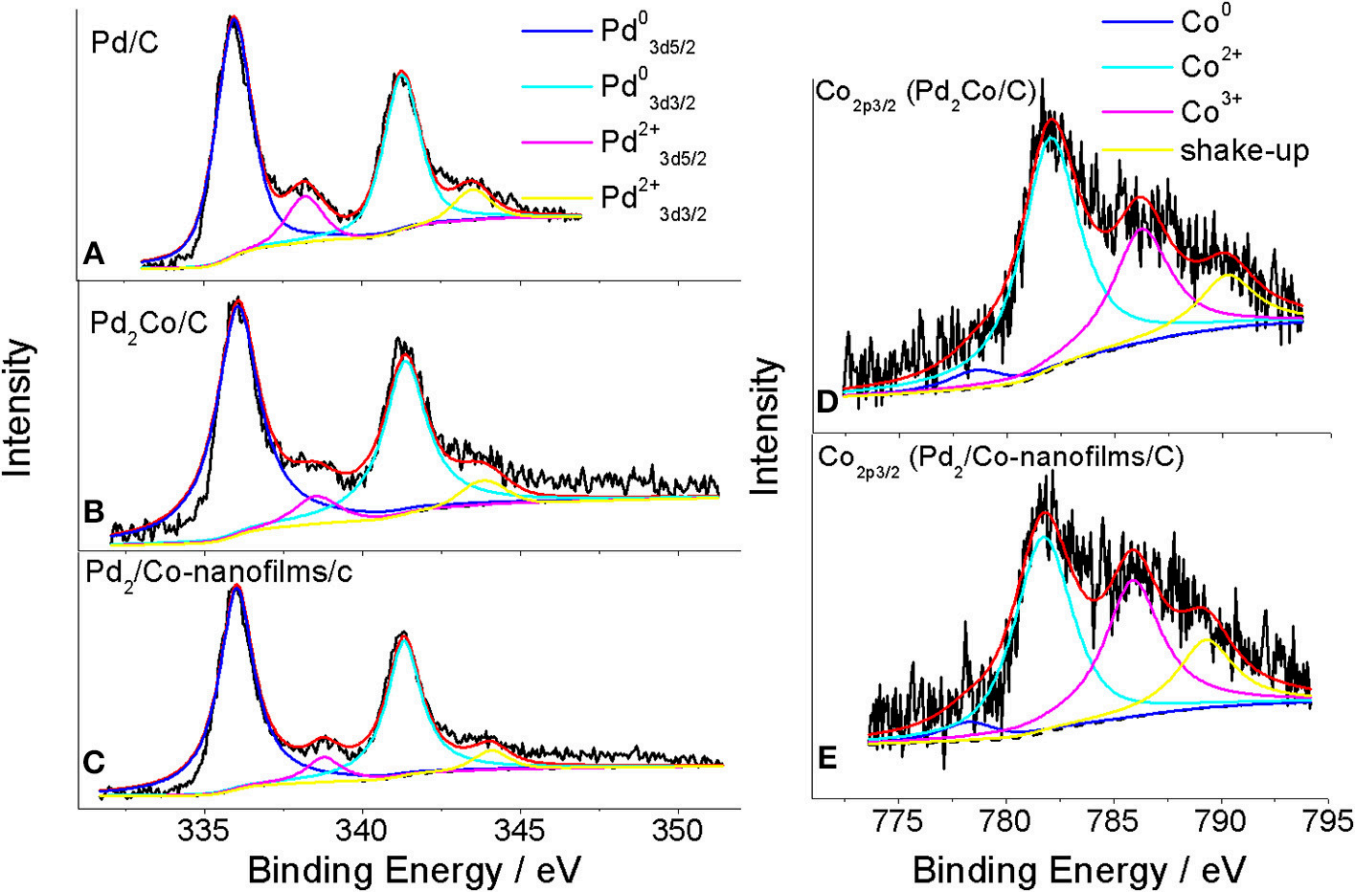
XPS spectra of Pd_32_d **(A)** for Pd/C, Pd_3_d **(B,C)** and Co_2_p **(D,E)** for Pd_2_Co/C and Pd_2_/Co-nanofilms/C catalysts. The black solid lines are the measured XPS responses. The black dashed lines are the background determined with the Shirley function. The red lines are the superposition of the deconvoluted component spectra and the background.

The chemical states of Pd and Co on the catalytic surface were examined by XPS. Figure 3 presents the XPS responses of the prepared Pd/C, Pd_2_Co/C and Pd_2_/Co-nanofilms/C catalysts. The Pd states for all studied samples consist of Pd metal (Pd^0^) as well as Pd oxide (Pd^2+^) by fitting the Pd 3d in the binding energy region between 330 and 352 eV using Gauss–Lorentzian fitting methods (Figures 3A–C) (Oishi and Savadogo, 2013; Stefanov et al., 2015; Yun et al., 2015). Different from Pd_2_Co/C catalyst with higher binding energy 336.06 eV for Pd 3d_5/2_ with respect to the value 335.96 eV for Pd/C as a result of the effect of alloying Pd with Co (Stefanov et al., 2015; Yun et al., 2015), the binding energy for Pd 3d_5/2_ of the prepared Pd_2_/Co-nanofilms/C catalyst was 336.02 eV medially. The corresponding peak areas can be used to estimate the relative Pd^0^/Pd^2+^ atom ratios in the near-surface region of each catalyst. The estimated near-surface Pd^0^/Pd^2+^ ratios are ~0.91 and 0.89 for the Pd_2_Co/C and Pd_2_/Co-nanofilms/C catalysts, respectively, which are much higher than the 0.55 ratio for Pd/C. These observations confirm the Co nanofilms-induced modification of electronic properties or geometric structure of Pd nanoparticles. The Co 2p_3/2_ XPS peaks in Figures 3D,E show that Co states of the exposed Co atoms consist of Co metal (Co^0^) and Co oxides (Co^2+^) (Ali et al., 2014; Yun et al., 2015). After the two shakeup satellite peaks associated with the Co (2p) line of Co^2+^ are taken into account (Xiao et al., 2015), the major Co 2p_3/2_ XPS peaks with the typical binding energy around 782 eV correspond to Co^2+^ oxides. This result suggests that a majority of the exposed Co atoms have been oxidized during post-processing. The alloying effect including the addition of an easily oxidized metal (e.g., Co or Ni), which can preferentially combine with oxygen to prevent the oxidation of noble metals, may explain the higher Pd^0^/Pd^2+^ ratios for Pd_2_Co/C and Pd_2_/Co-nanofilms/C catalysts calculated by XPS peak areas (Chen et al., 2011; Jiang et al., 2015). Although the Co nanofilm substrate cannot be resolved from the responses of TEM and XRD of the carbon supported sample, the measured results of Co nanofilms (Figure 1A, also see Chen et al., 2016) and the Co XPS profile of the Pd_2_/Co-nanofilms/C catalyst verify the successful fabrication of Co nanofilms.

### Cyclic voltammetric measurement

**Figure 4:**
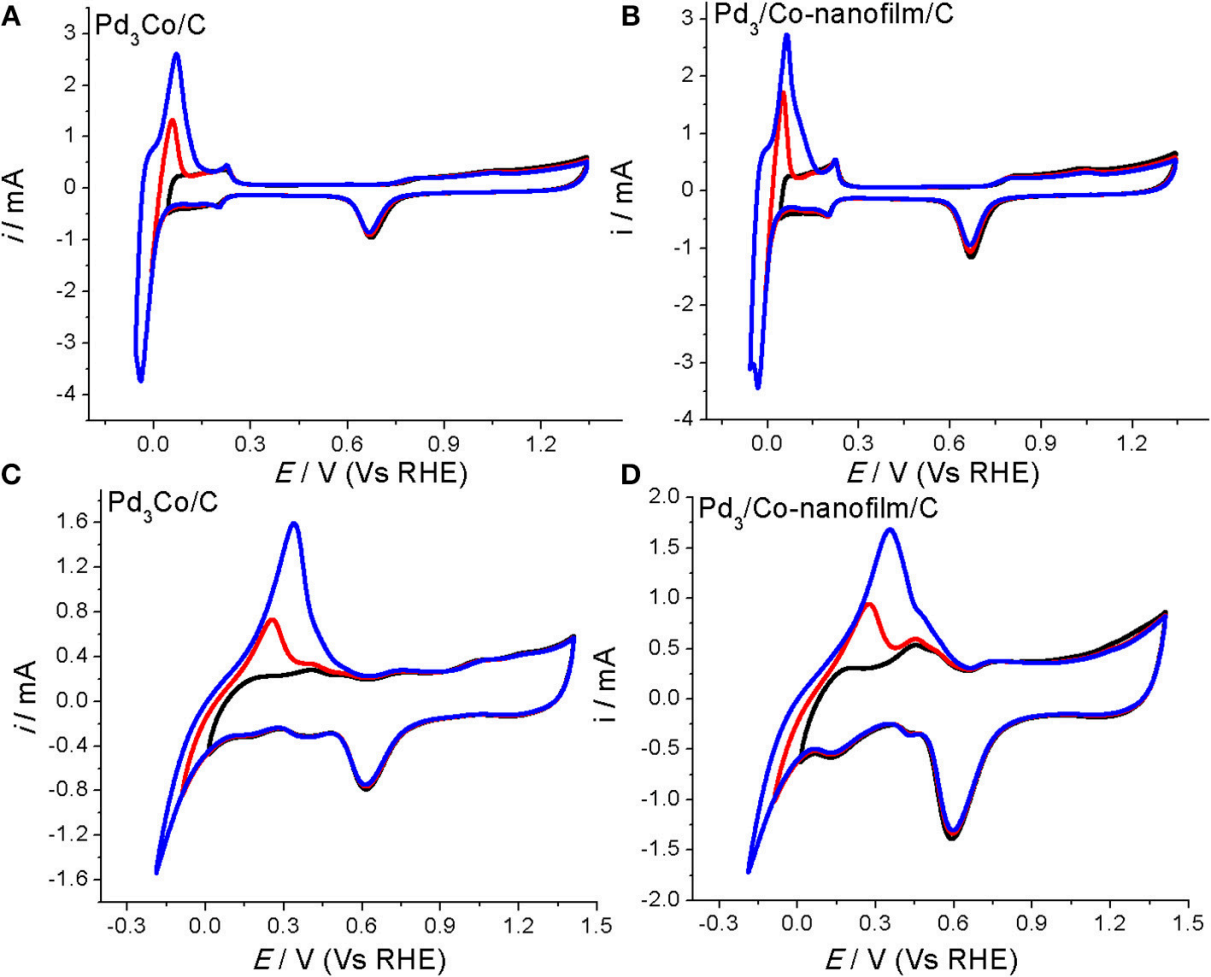
CVs of the prepared Pd_3_Co/C and Pd_3_/Co-nanofilms/C catalysts at room temperature 27°C in the different potential regions **(A,B)** in Ar-saturated 0.5 M H_2_SO_4_ and **(C,D)** in Ar-saturated 0.1 M NaOH. Scanning rates: 50 mV/s.

A clean surface is important to study the characteristic and catalytic active area of an electrocatalysts. We tested the CVs repeatedly in the different potential regions by changing the cathodic scan potentials. Take the Pd-Co samples for example (Figure 4), the H adsorption/desorption reaction on the surface of Pd_3_Co/C and Pd_3_/Co-nanofilms/C catalysts under cathodic potential (0.0103 V (Vs RHE) becomes the main reaction in 0.1 M NaOH solution, in which H absorption peak cannot take place. Similarly, H absorption peak is suppressed under cathodic potential [0.042 V (Vs RHE)] in 0.5 M H_2_SO_4_. And the H absorption become the main process along with reducing the cathodic potentials, covering the adsorption/desorption peaks (seen the red and blue lines in Figure 4). Sun et al. studied the CVs on the nm-Pd film formed by the 6 nm Pd crystal and compared with those of the bulk Pd metal electrode in 0.5 M H_2_SO_4_ solution (Cai et al., 1999, Figures 4, 6 in the reported paper). The H adsorption/desorption reaction on the surface of nm-Pd electrode take place under lower cathodic potential [0.032 V (Vs RHE)] without H absorption. However, on the bulk Pd metal, the adsorption/desorption current peaks could be obviously observed under high cathodic scan potential [(0.172 V (Vs RHE)]. When the cathodic potential was reduced to [0.032 V (Vs RHE)], the H absorption becomes the main process covering the adsorption/desorption peaks. The fact that H dissolution into Pd causes larger peak in the hydrogen potential region is discussed in reported paper (Zhang et al., 2007; Lee et al., 2015). As for the CV scans in our measurement, the CV characteristics are consistent with those results reported. And the H adsorption/desorption reaction and H absorption process can be resolved clearly under different cathodic potential regions. Those results indicate a clean surface of Pd-based catalysts in our experiment.

**Figure 5:**
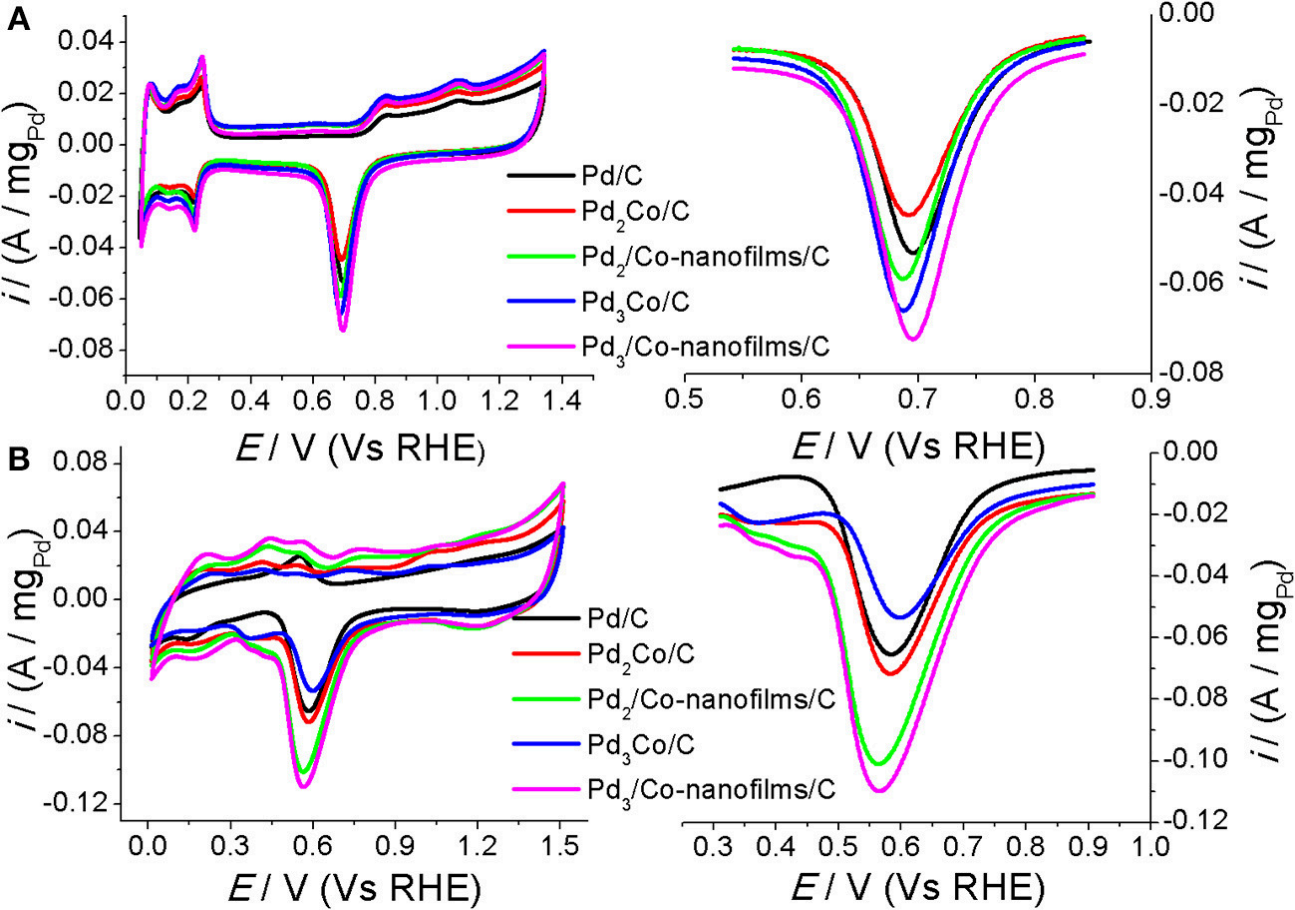
CVs of the prepared Pd/C, Pd_x_Co/C and Pd_x_/Co-nanofilms/C catalysts at room temperature 27°C and enlarged current peaks for the stripping of the oxygenated adsorbates **(A)** in Ar-saturated 0.5 M H_2_SO_4_ and **(B)** in Ar-saturated 0.1 M NaOH. Scanning rates: 50 mV/s.

Figure 5 gives the cyclic voltammograms (CVs) and the corresponding voltammetric peaks associated with the reduction desorption of the oxygenated adsorbates of the prepared Pd/C, Pd_x_Co/C, and Pd_x_/Co-nanofilms/C catalysts in argon-saturated H_2_SO_4_ solution (Figure 5A) and NaOH solution (Figure 5B). It can be seen that the composite Pd_x_/Co-nanofilms/C catalysts exhibit evidently larger electrochemical areas compared with the Pd_x_Co/C catalysts of the same Pd/Co ratios, particularly in alkaline media. The enlarged catalytic areas of Pd_x_/Co-nanofilms/C catalysts are attributed to the rough surface of Pd nanoparicles with a distorted crystal lattice measured from HRTEM and XRD (Figures 2, 3). In H_2_SO_4_ solution, the electrochemical areas increase with the addition of Pd content both for the Pd_x_Co/C alloy and Pd_x_/Co-nanofilms/C catalysts. However, in NaOH solution, the Pd_x_Co/C alloy catalysts cannot release all of the active sites and the catalytic areas are not absolutely dependent on Pd content, such as more difference of electrochemical areas between the Pd_x_Co/C and the Pd_x_/Co-nanofilms/C catalysts with the same Pd/Co atom ratios and the Pd_3_Co/C catalyst exhibits the smallest active area. These characteristics may be related to the existence form of Co atoms in Pd-based materials and the electrolyte properties. In alkaline media, exposed Co atoms are oxidized in the electrochemical scans. The Co oxidation near Pd in Pd_x_Co alloy may cover some active sites of surface Pd, corresponding to the reduced active areas. However, in the designed composite structure of Pd/Co-nanofilms, the oxidation of segregated Co nanofilm substrate may almost not affect the surface active centers of Pd nanoparticles. The variation in peak potentials for the reduction desorption of the oxygenated adsorbates (*E*_o_) is inconsistent in acid and alkaline media, indicated by the dashed dividing lines through the peak position of Pd/C. The results predict the different regular patterns of ORR activity in acid and alkaline media.

### ORR activity and stability measurements

**Figure 6:**
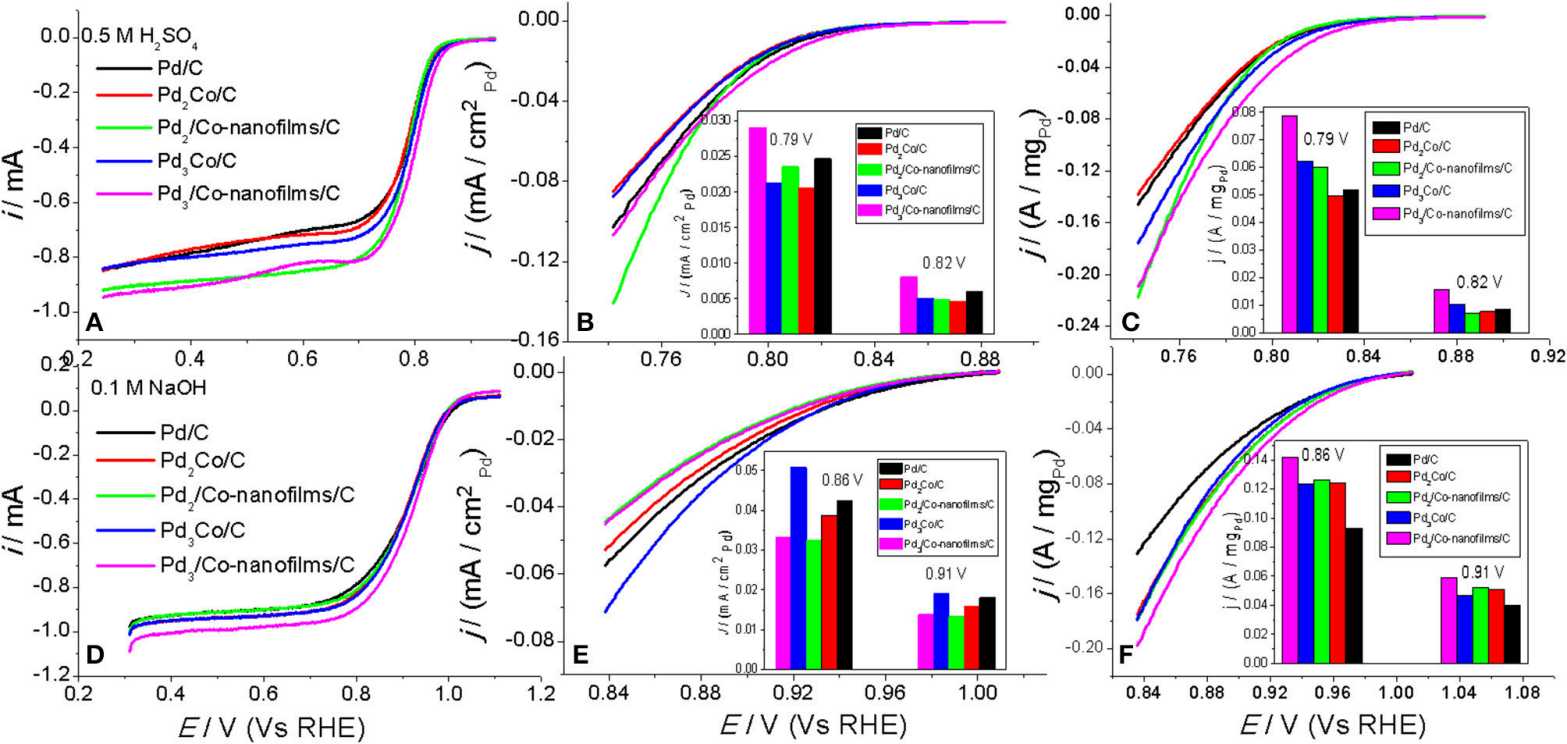
ORR polarization curves for the prepared Pd/C, Pd_x_Co/C, and Pd_x_/Co-nanofilms/C catalysts, the corresponding specific area and mass activities mass activities of Pd measured in 0.5 M H_2_SO_4_ **(A–C)** and in 0.1 M NaOH **(D–F)**, respectively. Inset of **(B,C,E,F)**: the comparison of mass activity under different potentials.

The electrocatalytic ORR performances of the prepared Pd_x_/Co-nanofilms/C catalysts are investigated using a rotating disk electrode at a rotation rate of 1,600 rpm in O_2_-saturated 0.5 M H_2_SO_4_ and 0.1 M NaOH electrolyte, respectively. We technically repeat the experiment 3–5 times to get consistent results. Figures 6A,D show the ORR polarization curves, in which the curves for the prepared Pd/C and Pd_x_Co/C alloy catalysts are also given for comparison. The Pd specific area and mass activities, which are acquired by normalizing the kinetic current (*I*_k_) by the electrochemical surface area of Pd (ECSA, determined from the oxygen discharges in CVs given in Figure 5) and by the Pd loading on the electrode, are presented in Figures 6B,C,E,F (the inset). The *I*_k_ was calculated from the steady-state polarization curves (Figures 6A,D) using Koutecky–Levich equation (*I*_k_ = *I* × *I*_L_/(*I*_L_-*I*)), where *I* and *I*_L_ are the measured current and the corresponding limiting diffusion current, respectively.

The steady-state polarization curves display a diffusion-limiting current region (from 0.242 to 0.742 V in H_2_SO_4_ solution and from 0.31 to 0.81 V in NaOH solution) and a mixed kinetic-diffusion control region (from 0.742 to 0.842 V in H_2_SO_4_ solution and from 0.810 to 1.010 V in NaOH solution). The half-wave potential (*E*_1/2_) of the Pd_3_/Co-nanofilms/C (0.799 V) is higher than that of Pd/C (0.782 V) and Pd_3_Co/C alloy (0.788 V) catalysts in acid solution. This regular pattern is also found in alkaline solution with *E*_1/2_ values of 0.910, 0.892, and 0.896 V for the Pd_3_/Co-nanofilms/C, Pd/C, and Pd_3_Co/C alloy catalysts, respectively. The Pd/C ORR activity is equal to that of reported paper (Wang et al., 2017). The Pd specific area activity profile in acid solution (Figure 6B) shows slightly enhanced activities of the Pd_x_/Co-nanofilms/C catalysts compared with Pd_x_Co/C catalysts. However, in alkaline solution (Figure 6E) the specific activities show the exact opposite regular. Maybe in the acid solution the surface Pd atoms exist in pure Pd along with accelerated Co dissolution. Such, the Pd_x_Co/C alloy catalysts cannot display the enhanced specific activity. While, in alkaline solution the Pd_x_Co/C alloying effect enhances Pd intrinsic activity (Yun et al., 2015; Bampos et al., 2017). As indicated by the mass activity profiles (Figures 6C,F) and the corresponding comparison under given potentials among the prepared catalysts, the Pd_3_/Co-nanofilms/C catalyst shows the highest mass activity in both acid and alkaline solution. The results reveal that the Pd_x_/Co-nanofilms/C catalysts possess a superior catalytic performance. Previous theoretical and experimental works are focus on the methods to down-shift the d-band center of noble metals (e.g., Pd and Pt) by alloying a precious metal with the transition metal with a smaller interatomic distance (e.g., alloy structure, core-shell structure, and so on), thereby weakening the adsorption of oxygenated intermediates and being beneficial to improve ORR activity (Wei et al., 2011; Jiang et al., 2015; Yun et al., 2015). In fact, it is well know that the catalytic process happens on the surface of the catalysts. Then a large specific surface area is an effective factor to reduce the amount of the precious metal. The significantly increased catalytic area of the Pd_x_/Co-nanofilms/C catalysts (showed by CV scans in Figure 5) should be the reason why this structure exhibit enhanced ORR activity compared with Pd_x_Co/C alloy and Pd/C catalysts. Besides, Pd surface roughness and oxidation degree in the electrochemical process can modify the ORR activity.

As shown in Figures 6C,F, the mass activity of the Pd_x_/Co-nanofilms/C catalysts exhibit different dependence on the Pd/Co atom ratios in acid and alkaline media. The Pd_x_Co/C alloy catalysts also display the same behavior. In 0.5 M H_2_SO_4_ solution, the ORR mass activities of Pd improve remarkably with increasing the Pd/Co atom ratios. However, the mass activities are less dependent on the Pd/Co atom ratios in 0.1 M NaOH solution. These regular patterns predict that appropriate addition of Pd content is beneficial to improve ORR activity both in acid and alkaline media, particularly in former media.

**Figure 7:**
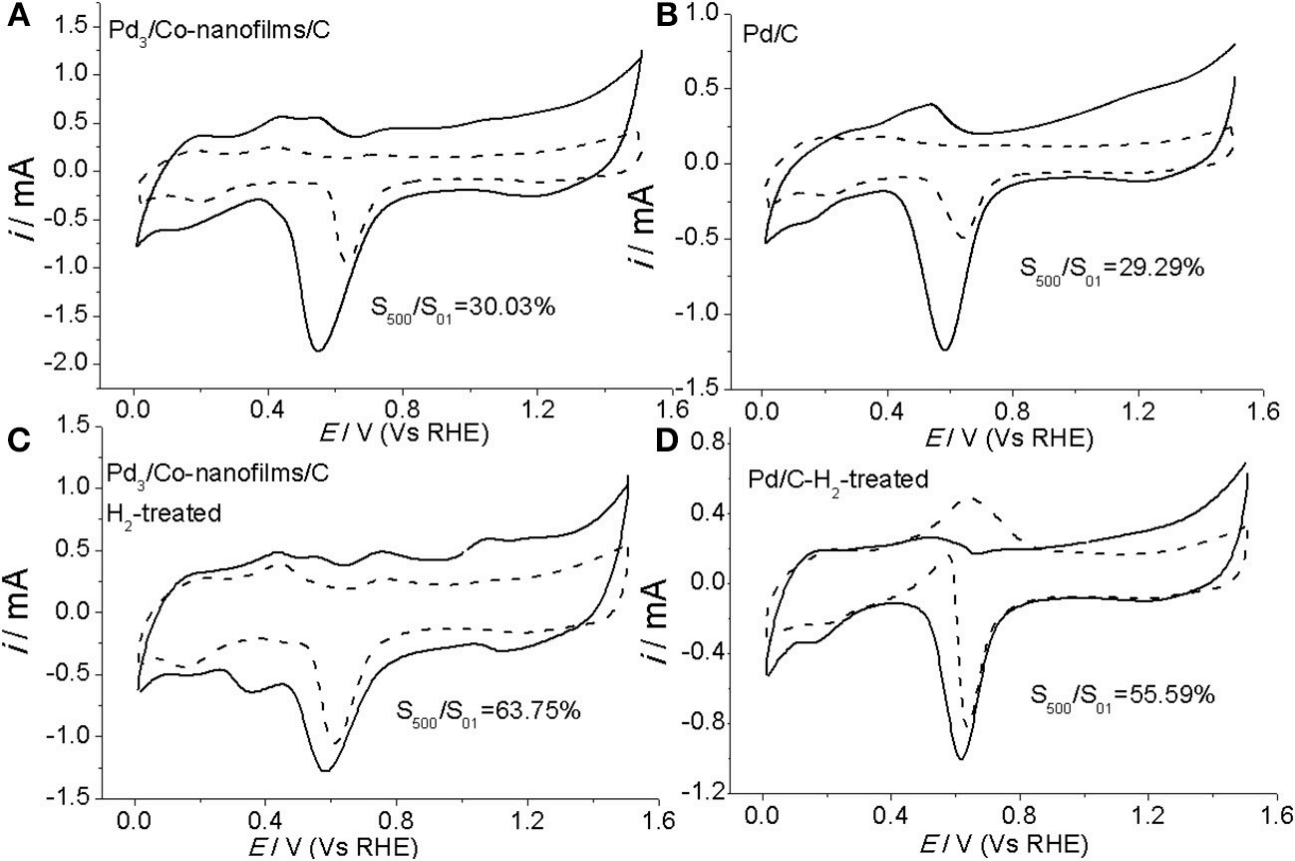
CVs of Pd_3_/Co-nanofilms/C **(A,C)** and Pd/C catalysts **(B,D)** before (solid) and after (dashed) accelerated by electrochemical degradation tests under repeating potential cycling in 0.1 M NaOH.

Except for the catalytic activity, the long-term stability is another important parameter that determines the practical application of a specific ORR electrocatalyst. The stability of the prepared Pd_3_/Co-nanofilms/C and pure Pd/C catalysts are evaluated by measuring their electrochemical surface areas from the desorption of the oxygenated adsorbates after extended potential cycling under the accelerated potential region between 0.0103 and 1.510 V (Vs RHE) in Ar-saturated 0.1 M NaOH with a scan rate of 50 mV/s. Figure 7 gives the initial and final CVs after 500 cycles of the Pd_3_/Co-nanofilms/C and Pd/C catalysts before and after heat-treatment in H_2_ atmosphere. The stability of the Pd_3_/Co-nanofilms/C catalyst is comparable with that of pure Pd/C catalyst before heat-treatment, in which self-existent Co-nanofilm substrate do not cause the violent degradation of the catalytic area (Figures 7A,B), owing to relatively lower corrosion of transitional metal (e.g., Co and Ni) in alkaline media and the enhanced effect by the surface Pd nanoparticles. After proper heat-treatment in H_2_ atmosphere at 200°C, both Pd_3_/Co-nanofilms/C and Pd/C catalysts exhibit decreased variations in potential CVs after long-time potential cycling. Moreover, the Pd_3_/Co-nanofilms/C catalyst exhibits greater cycling stability than pure Pd/C catalyst. One of the reasons for the relatively superior stability of the prepared Pd_3_/Co-nanofilms/C after heat-treatment might be due to the higher coordination of surface Pd atoms. In addition, the heat-treatment promotes a little amount of Co atoms into Pd crystal lattice to increase atomic bond strength, which can further enhance stability of the Pd_3_/Co-nanofilms/C catalyst.

## Conclusion

Applying the interfacial synthesis technique on an immiscible ionic liquid (IL)/water interface, Pd nanoparticles with an average size of 5 nm are implanted onto 2D Co-nanofilm substrate to form the composite Pd_x_/Co-nanofilms/C catalysts. The TEM (HRTEM), XRD, XPS, and CVs results suggest that the assembly of Pd atoms may be affected by the Co nanofilms, in which the Pd nanoparticles show a marked distortion of crystal lattice and surface roughness. These Pd_x_/Co-nanofilms/C electrocatalysts exhibit enhanced activity for ORR compared with Pd/C and Pd_x_Co/C catalysts in both acid and alkaline solutions, in which the Pd_3_/Co-nanofilms/C catalyst displays the highest ORR mass activity. The superior ORR mass activities of the fabricated Pd_x_/Co-nanofilms/C catalysts may be attributed to their larger catalytic areas, which are conferred by the rough surface of Pd nanoparticles with a distorted crystal lattice, and the synergistic effect between the surface Pd atoms and the 2D Co nanofilm substrate. Furthermore, by using proper heat-treatment methods, the Pd_x_/Co-nanofilms/C catalysts exhibit improved cycling stability compared with pure Pd/C catalyst after extended potential cycling. This study can predict that deposition of Pd-M alloy nanopartices onto 2D transition metal nanofilms would further optimize the electrochemical activity and stability of Pd-based catalysts.

## Author contributions

YC and BL designed experiments. LA carried out experiments. JS carried out the electrochemical experimental. YC, JC, and JY analyzed experimental results. LA and YC wrote the manuscript.

### Conflict of interest statement

The authors declare that the research was conducted in the absence of any commercial or financial relationships that could be construed as a potential conflict of interest.

## References

[B1] Ali M. Witkowska A. Abbas M. Gunnella R. Di Cicco A. (2014). Evolution of the nanostructure of Pt and Pt-Co polymer electrolyte membrane fuel cell electrocatalysts at successive degradation stages probed by X-ray photoemission. J. Power Sources 271, 548–555. 10.1016/j.jpowsour.2014.08.028

[B2] Antolini E. (2014). Effect of structural characteristics of binary palladium-cobalt fuel cell catalysts on the activity for oxygen reduction. ChemPlusChem 79, 765–775. 10.1002/cplu.201402034

[B3] Arroyo-Ramírez L. Montano-Serrano R. Luna-Pineda T. Román F. R. Raptis R. G. Cabrera C. R. (2013). Synthesis and characterization of palladium and palladium cobalt nanoparticles on vulcan XC-72R for the oxygen reduction reaction. ACS Appl. Mater. Inter. 5, 11603–11612. 10.1021/am402932h 24102312

[B4] Bampos G. Bebelis S. Kondarides D. I. Verykios X. (2017). Comparison of the activity of Pd-M (M: Ag, Co, Cu, Fe, Ni, Zn) bimetallic electrocatalysts for oxygen reduction reaction. Top. Catal. 60, 1269–1273. 10.1007/s11244-017-0795-z

[B5] Cai L. R. Sun S. G. Xia S. Q. Chen F. Zheng M. S. Chen S. P. (1999). Particular properties and structure of electrochemically prepared Pd film nano-materials. Acta Phys. Chem. Sin. 15, 1023–1029. 10.3866/Pku.Whxb19991113

[B6] Chen Y. M. Chen M. M. Shi J. C. Yang J. Fan Y. P. (2016). Pd nanoparticles on Co nanofilms as composite electrocatalysts for ethanol oxidation in alkaline solution. Int. J. Hydrogen Energy 41, 17112–17117. 10.1016/j.ijhydene.2016.07.105

[B7] Chen Y. M. Chen M. M. Shi J. C. Yang J. Zhang D. F. (2014). Fabrication of “clean” nano-structured metal materials on ionic liquid/water interface. Mater. Lett. 132, 153–156. 10.1016/j.maltet.2014.06.052

[B8] Chen Y. M. Liang Z. X. Yang F. Liu Y. W. Chen S. L. (2011). Ni–Pt Core–Shell nanoparticles as oxygen reduction electrocatalysts: effect of Pt shell coverage. J. Phys. Chem. C 115, 24073–24079. 10.1021/jp207828n

[B9] Cheng H. Chen J. M. Li Q. J. Su C. Y. Chen A. N. Zhang J. X.. (2017a). A modified molecular framework derived highly efficient Mn-Co-carbon cathode for flexible Zn-air battery. Chem. Comm. 53, 11596–11599. 10.1039/C7CC04099G 28991305

[B10] Cheng H. Li M. L. Su C. Y. Li N. Liu Z. Q. (2017b). Cu-Co bimetallic oxide quantum dot decorated nitrogen-doped carbon nanotubes: a high-efficiency bifunctional oxygen electrode for Zn–air batteries. Adv. Funct. Mater. 27:1701833 10.1002/adfm.201701833

[B11] Doan T. T. V. Wang J. Poon K. C. Tan D. C. Khezri B. Webster R. D. (2016). Theoretical modelling and facile synthesis of a highly active boron-doped palladium catalyst for the oxygen reduction reaction. Angew. Chem. Int. Edit. 55, 6842–6847. 10.1002/anie.201601727 27086729

[B12] Gong Y. X. Wang Y. Sun G. Jia T. K. Jia L. Zhang F. M. (2018). Carbon nitride decorated ball-flower like Co_3_O_4_ hybrid composite: hydrothermal synthesis and ethanol gas sensing application. Nanomaterials 8:132 10.3390/nano8030132 PMC5869623 29495469

[B13] He J. L. He Y. Z. Fan Y. N. Zhang B. Du Y. C. Wang J. Y. (2017). Conjugated polymer-mediated synthesis of nitrogen-doped carbon nanoribbons for oxygen reduction reaction. Carbon 124, 630–636. 10.1016/j.carbon.2017.08.08

[B14] Holade Y. da Silva R. G. Servat K. Napporn T. W. Canaff C. de Andrade A. R. (2016). Facile synthesis of highly active and durable PdM/C (M = Fe, Mn) nanocatalysts for the oxygen reduction reaction in an alkaline medium. J. Mater. Chem. A 4, 8337–8349. 10.1039/c6ta02096h

[B15] Huang B. Chen L. Wang Y. Ouyang L. Ye J. (2017). Paragenesis of palladium-cobalt nanoparticle in nitrogen-Rich carbon nanotubes as a bifunctional electrocatalyst for hydrogen-evolution reaction and oxygen reduction reaction. Chem. Eur. J. 23, 7710–7718. 10.1002/chem.201700544 28258967

[B16] Jiang G. Zhu H. Zhang X. Shen B. Wu L. Zhang S.. (2015). Core/shell face-centered tetragonal FePd/Pd nanoparticles as an efficient non-Pt catalyst for the oxygen reduction reaction. ACS Nano 9, 11014–11022. 10.1021/acsnano.5b04361 26434498

[B17] Kuttiyiel K. A. Sasaki K. Su D. Wu L. Zhu Y. Adzic R. R. (2014). Gold-promoted structurally ordered intermetallic palladium cobalt nanoparticles for the oxygen reduction reaction. Nat. Commun. 5, 51–85. 10.1038/ncomms6185 25373826

[B18] Lee K. Savadogo O. Ishihara A. Mitsushima S. Kamiya N. Ota K. (2015). Methanol-tolerant oxygen reduction electrocatalysts based on Pd-3D transition metal alloys for direct methanol fuel cells. J. Electrochem. Soc. 153, A20–A24. 10.1149/1.2128101

[B19] Li J. C. Wu X. T. Chen L. J. Li N. Liu Z. Q. (2018). Bifunctional MOF-derived Co-N-doped carbon electrocatalysts for high-performance zinc-air batteries and MFCs. Energy 156, 95–102. 10.1016/j.energy.2018.05.096

[B20] Li Z. Ji S. Pollet B. G. Shen P. K. (2014). A Co_3_W_3_C promoted Pd catalyst exhibiting competitive performance over Pt/C catalysts towards the oxygen reduction reaction. Chem. Commun. 50, 566–568. 10.1039/c3cc48240e 24271116

[B21] Liu S. Zhang Q. Li Y. Han M. Gu L. Nan C.. (2015a). Five-fold twinned Pd2NiAg nanocrystals with increased surface Ni site availability to improve oxygen reduction activity. J. Am. Chem. Soc. 137, 2820–2823. 10.1021/ja5129154 25626352

[B22] Liu Z. T. Huang K. L. Wu Y. S. Lyu Y. P. Lee C. L. (2015b). A comparison of physically and chemically defective graphene nanosheets as catalyst supports for cubic Pd nanoparticles in an alkaline oxygen reduction reaction. Electrochim. Acta 186, 552–561. 10.1016/j.electacta.2015.11.025

[B23] Lu Y. Jiang Y. Gao X. Wang X. Chen W. (2014). Strongly coupled Pd nanotetrahedron/tungsten oxide nanosheet hybrids with enhanced catalytic activity and stability as oxygen reduction electrocatalysts. J. Am. Chem. Soc. 136, 11687–11697. 10.1021/ja5041094 25054583

[B24] Maheswari S. Sridhar P. Pitchumani S. (2013). Pd–TiO2/C as a methanol tolerant catalyst for oxygen reduction reaction in alkaline medium. Electrochem. Commun. 26, 97–100. 10.1016/j.elecom.2012.10.021

[B25] Oishi K. Savadogo O. (2013). Correlation between the physico-chemical properties and the oxygen reduction reaction electro catalytic activity in acidic medium of Pd–Co alloys synthesized by ultrasonic spray method. Electrochim. Acta 98, 225–238. 10.1016/j.electacta.2013.02.033

[B26] Poon K. C. Tan D. C. Vo T. D. Khezri B. Su H. Webster R. D.. (2014). Newly developed stepwise electroless deposition enables a remarkably facile synthesis of highly active and stable amorphous Pd nanoparticle electrocatalysts for oxygen reduction reaction. J. Am. Chem. Soc. 136, 5217–5220. 10.1021/ja500275r 24661048

[B27] Rahul R. Singh R. K. Neergat M. (2014). Effect of oxidative heat-treatment on electrochemical properties and oxygen reduction reaction (ORR) activity of Pd-Co alloy catalysts. J. Electroanal. Chem. 712, 223–229. 10.1016/j.jelechem.2013.11.011

[B28] Shao M. Chang Q. Dodelet J. P. Chenitz R. (2016). Recent advances in electrocatalysts for oxygen reduction reaction. Chem. Rev. 116, 3594–3657. 10.1021/acs.chemrev.5b00462 26886420

[B29] Song K. P. Zou Z. J. Wang D. L. Tan B. E. Wang J. Y. Chen J. (2016). Microporous organic polymers derived microporous carbon supported Pd catalysts for oxygen reduction reaction: impact of framework and heteroatom. J. Phys. Chem. C 120, 2187–2197. 10.1021/acs.jpcc.5b10358

[B30] Srejic I. Rakocevic Z. Nenadovic M. Strbac S. (2015). Oxygen reduction on polycrystalline palladium in acidic and alkaline solutions: topographical and chemical Pd surface changes. Electrochim. Acta 169, 22–31. 10.1010/j.electacta.2015.04.032

[B31] Stefanov P. Todorova S. Naydenov A. Tzaneva B. Kolev H. Atanasova G. (2015). On the development of active and stable Pd–Co/c-Al_2_O_3_ catalyst for complete oxidation of methane. Chem. Eng. J. 266, 329–338. 10.1016/j.cej.2014.12.099

[B32] Su C. Y. Cheng H. Li W. Liu Z. Q. Li N. Hou Z. F. (2017). Atomic modulation of FeCo–nitrogen–carbon bifunctional oxygen electrodes for rechargeable and flexible all-solid-state zinc–air battery. Adv. Energy Mater. 7:20160242 10.1002/aenm.20160242

[B33] Sui S. Wang X. Zhou X. Su Y. Riffat S. Liu C. A. (2017). Comprehensive review of Pt electrocatalysts for the oxygen reduction reaction: nanostructure, activity, mechanism and carbon support in PEM fuel cells. J. Mater. Chem. A 5, 1808–1825. 10.1039/c6ta08580f

[B34] Wang G. W. Guan J. X. Xiao L. Huang B. Wu N. Lu J. T. (2016). Pd skin on AuCu intermetallic nanoparticles: a highly active electrocatalyst for oxygen reduction reaction in alkaline media. Nano Energy 29, 268–274. 10.1016/j.nanoen.2016.04.005

[B35] Wang J. Y. Xu M. Zhao J. Q. Fang H. F. Huang Q. Z. Xiao W. P. (2018). Anchoring ultrafine Pt electrocatalysts on TiO_2_-C via photochemical strategy to enhance the stability and efficiency for oxygen reduction reaction. Appl. Catal. B Environ. 237, 228–236. 10.1016/j.apcatb.2018.05.085

[B36] Wang M. Qin X. Jiang K. Dong Y. Shao M. Cai W. B. (2017). Electrocatalytic activities of oxygen reduction reaction on Pd/C and Pd–B/C catalysts. J. Phys. Chem. C 121, 3416–3423. 10.1021/acs.jpcc.6b12026

[B37] Wang Y. Huang W. Si C. H. Zhang J. Yan X. J. Jin C. H. (2016). Self-supporting nanoporous gold-palladium overlayer bifunctional catalysts toward oxygen reduction and evolution reactions. Nano Res. 9, 3781–3794. 10.1007/s12274-016-1248-x

[B38] Wei Y. C. Liu C. W. Lee H. W. Chung S. R. Lee S. L. Chan T. S. (2011). Synergistic effect of Co alloying and surface oxidation on oxygen reduction reaction performance for the Pd electrocatalysts. Int. J. Hydrogen Energ. 36, 3789–3802. 10.1016/j.ijhydene.2010.12.098

[B39] Xiao L. Zhuang L. Liu Y. Lu J. T. Abruña H. D. (2009). Activating Pd by morphology tailoring for oxygen reduction. J. Am. Chem. Soc. 131, 602–608. 10.1021/ja8063765 19108685

[B40] Xiao Y. H. Zhan G. H. Fu Z. G. Pan Z. C. Xiao C. M. Wu S. K. (2015). Titanium cobalt nitride supported platinum catalyst with high activity and stability for oxygen reduction reaction. J. Power Sources 284, 296–304. 10.1016/j.jpowsour.2015.03.001

[B41] Yan Z. X. Zhang M. M. Xie J. M. Zhu J. J. Shen P. K. (2015). A bimetallic carbide Fe_2_MoC promoted Pd electrocatalyst with performance superior to Pt/C towards the oxygen reduction reaction in acidic media. Appl. Catal. B Environ. 165, 636–641. 10.1016/j.apcatb.2014.10.070

[B42] Yun M. Ahmed M. S. Jeon S. (2015). Thiolated graphene oxide-supported palladium cobalt alloyed nanoparticles as high performance electrocatalyst for oxygen reduction reaction. J. Power Sources 293, 380–387. 10.1016/j.jpowsour.2015.05.094

[B43] Zhang L. Lee K. Zhang J. (2007). The effect of heat treatment on nanoparticle size and ORR activity for carbon-supported Pd-Co alloy electrocatalysts. Electrochim. Acta 52, 3088–3094. 10.1016/j.electacta.2006.09.051

[B44] Zhang L. L. Chang Q. W. Chen H. M. Shao M. H. (2016). Recent advances in palladium-based electrocatalysts for fuel cell reactions and hydrogen evolution reaction. Nano Energy 29, 198–219. 10.1016/j.nanoen.2016.02.044

[B45] Zhen Y. Lin L. L. Ma D. (2014). Construction of Pd-based nanocatalysts for fuel cells: opportunities and challenges. Catal. Sci. Technol. 4, 4116–4128. 10.1039/c4cy00760c

[B46] Zheng Y. Zhao S. Liu S. Yin H. Chen Y. Y. Bao J.. (2015). Component- controlled synthesis and assembly of Cu-Pd nanocrystals on graphene for oxygen reduction reaction. ACS Appl. Mater. Inter. 7, 5347–5357. 10.1021/acsami.5b01541 25695756

